# Customized 3D printed helmet in the treatment of metopic craniosynostosis in a 7-month-old infant, a case report

**DOI:** 10.3389/fped.2025.1474412

**Published:** 2025-03-14

**Authors:** Huthaifa Atallah, Rabee Naeem, Amneh Alshawabka, Anas S. Said, Huda Alfatafta, Evelin Derkács, Dorottya Varga, Bálint Molics

**Affiliations:** ^1^ Prosthetics and Orthotics Department, School of Rehabilitation Sciences, The University of Jordan, Amman, Jordan; ^2^ Digital Fabrication Division, Revolutionary Technologies for Medical Solutions, Amman, Jordan; ^3^ Neurosurgery Department, Al Basheer Hospital, Amman, Jordan; ^4^ Doctoral School of Health Sciences, Faculty of Health Sciences, University of Pécs, Pécs, Hungary; ^5^ Department of Sport Physiotherapy, Faculty of Health Sciences, University of Pécs, Pécs, Hungary

**Keywords:** 3D printing, helmet, metopic, craniosynostosis, case report

## Abstract

**Introduction:**

Metopic craniosynostosis results in a deformed skull and hence, impacts brain growth and development. Surgery is usually applied to treat this trigonocephalic head malformation. Helmet therapy is also utilized in craniosynostosis treatment after the surgery. However, data on use of 3D printed helmets in treatment of metopic craniosynostosis is lacking. Most of the studies are published about molding helmets. Moreover, there is a lack of evidence on its clinical outcomes. Therefore, the aim of this study was to assess the use of a 3D printed helmet in treating a metopic craniosynostosis, after the endoscopy-assisted craniotomy surgical intervention.

**Case description:**

A 7-month-old infant who was diagnosed with metopic craniosynostosis was referred from the neurosurgeon for a custom-made 3D printed helmet, after a surgical intervention. A certified orthotist has performed further assessment, scanning, designing, and printing a customized 3D printed helmet. Thereafter, fitting and delivery were successfully completed. Patient has come for two follow-up appointments, at 2 and 5 months.

**Results:**

Five months after the initial fitting, the head shape correction and reduction of deformity were noticed through anthropometric measures. The cranial vault asymmetrical index (CVAI) decreased from 7% to 2% and the cranial vault asymmetry (CVA) reduced from 7 mm to 3 mm.

**Conclusion:**

This case report illustrates the utilization of 3D printing technology in the treatment of metopic craniosynostosis. 3D printed helmets may offer an appropriate option for treating selective infants with metopic craniosynostosis. Thus, would introduce the 3D helmet as a following intervention for such cases after the endoscopy-assisted craniotomy surgical intervention. Further studies with a higher number of cases are compulsory to assess the effectiveness of treating metopic craniosynostosis by 3D printed helmets instead of molding helmets.

## Introduction

The metopic suture extends anteriorly from the nose to the sagittal suture at the top of the skull. It is the first skull suture to close physiologically, starting as early as 3 months (1–3). Premature fusion of the metopic suture in infants results in a condition called metopic craniosynostosis. Generally, when there is premature fusion of the sutures, the skull growth in that direction will be restricted, resulting in a continued growth perpendicular to other sutures that are open. This results in a deformed skull. A newborn with metopic craniosynostosis typically has a head that is formed like a triangle, with the widest half in the rear and the narrowest part in the front (4). Consequently, this trigonocephalic head malformation can impact brain growth and development. Severity of metopic craniosynostosis may vary and can result in long term complications. It can range from increase in intracranial pressure to neurodevelopment delays (2). Children with metopic craniosynostosis have been shown to be linked with the highest percentage of neurodevelopmental problems among all the single suture synostoses. Intellectual disability has been reported to be twice as high (4.8%) in children with metopic craniosynostosis as compared to those with sagittal or coronal synostosis (5). The incidence of metopic craniosynostosis varies widely, ranging from 1:700 to 1:15,000 newborns (6, 7). In the last few decades, its incidence has been rising, currently making it the second most common type of craniosynostosis (2).

Various treatment guidelines and studies have analysed the use of helmets especially in positional head deformities (8–17). Custom-made molding helmets are also used in various forms of craniosynostosis treatment; especially in patients undergoing endoscopy-assisted craniotomy surgery (18–25). To our knowledge, there is no study reporting the use of a 3D printed helmet with metopic craniosynostosis infants. Therefore, the purpose of this case study was to assess the use of a custom-made 3D printed helmet in treatment of metopic craniosynostosis, following an endoscopy-assisted craniotomy surgery. The cranial vault asymmetry (CVA) and cranial vault asymmetry index (CVAI) were calculated to evaluate the functionality of the 3D-printed helmet.

## Case description

**Figure 1:**
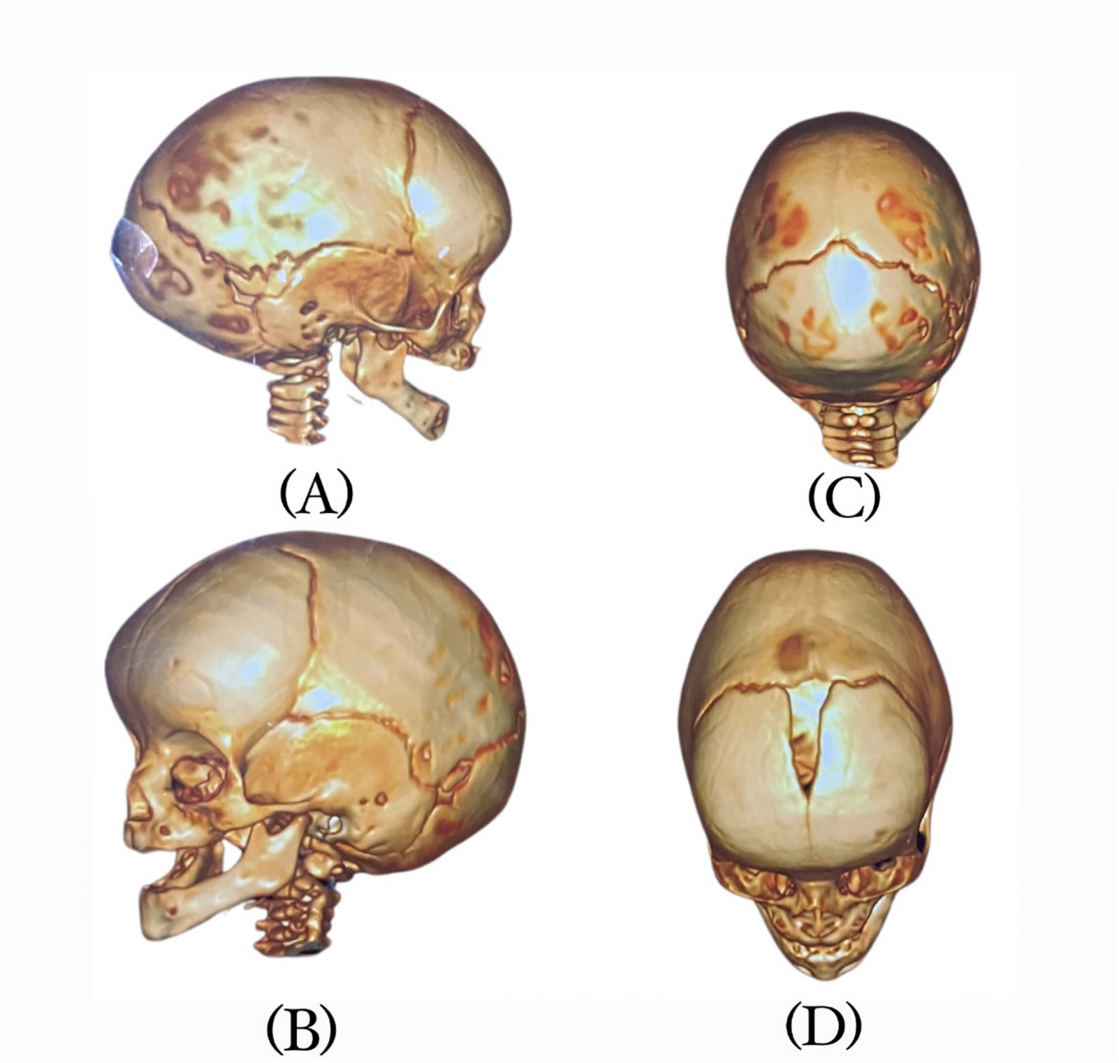
CT scan views of the infant's head: **(A)** right side, **(B)** left side, **(C)** superior, and **(D)** anterio-superior.

A 7-month-old infant was brought for initial physician assessment due to concerns of abnormal head symmetry. Birth and developmental history were insignificant. There was no intellectual disability and milestones were not delayed. She was born full term by normal vaginal delivery. There was no family history of craniosynostosis. History revealed that she was diagnosed with metopic craniosynostosis at the age of 5 weeks. Physical examination suggested closure of metopic suture. This was confirmed with radiological assessment by Computed tomography (CT) scan with 3D reconstruction (Figure 1). The patient was assessed by neurosurgery and an endoscopy-assisted craniotomy surgical intervention was applied at the age of 5 months. Unfortunately, and due to lack of certified orthotist with helmet experiences, she was referred to a certified orthotist for a custom-made helmet at the age of 7 months.

### Assessment

Ethical approval was obtained from the Ministry of Health (IRB-5680). Further orthotist assessment included measurements of head circumference, cranial diameters, palpation of the fontanelle and suture lines, and observational assessments of head shape, posturing and movements. Examination of head showed forward displacement of right ear, posterior flattening, and anterior bossing of the forehead (Figure 1). There was no abnormal head or neck posturing, rotation or tilting, and the range of motion was within normal. Multiple factors were considered in the decision to proceed with helmet therapy which was carried out after consulting with referring physician and parent's agreement. The main factors were age of the patient, moderate severity of deformity, skull pliability and parent's preferences. The first goal was to keep the abnormality from worsening. The second was to correct the deformity and restore the head to its natural shape as much as possible.

### Head scanning

**Figure 2:**
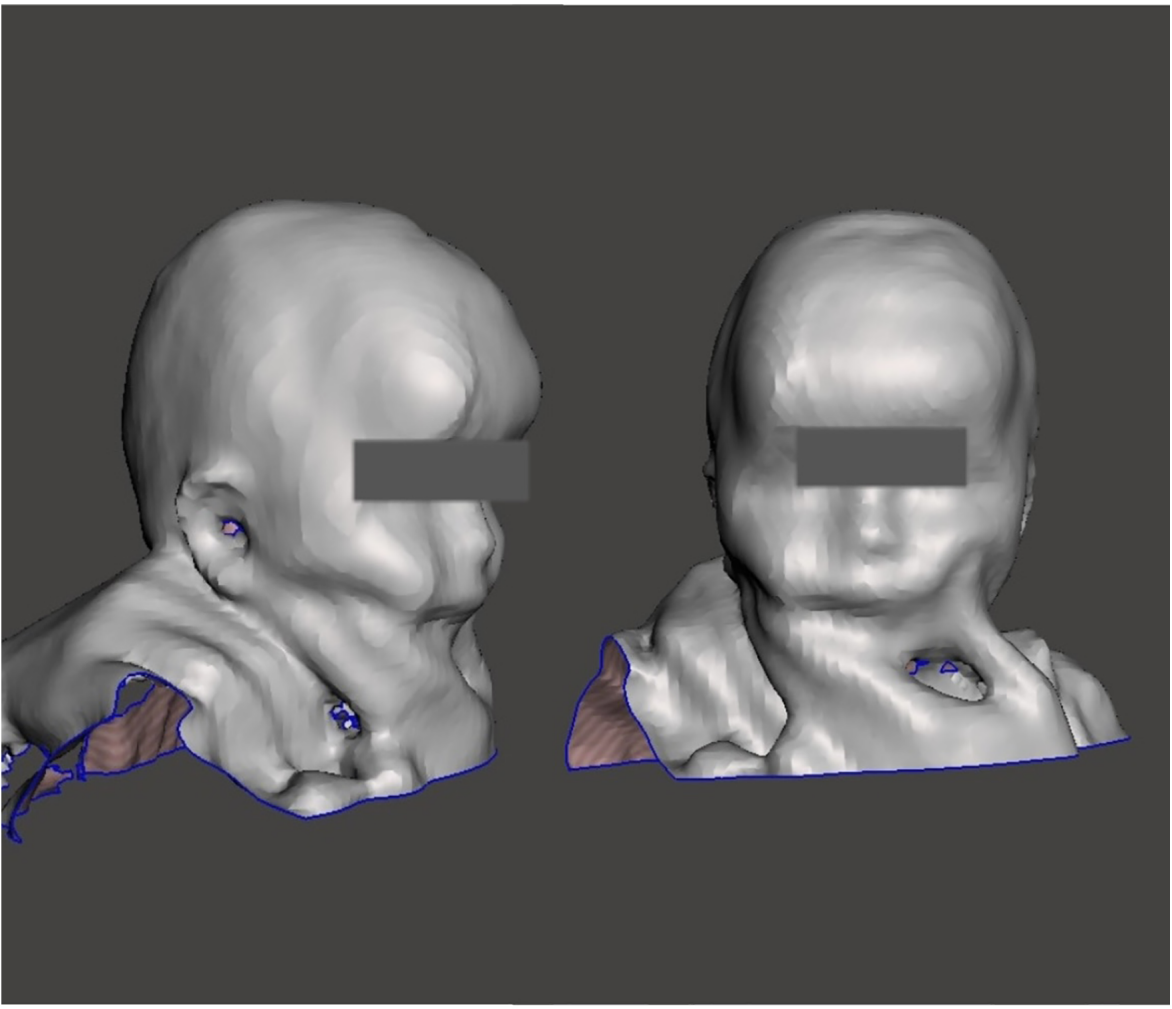
3D scan of the infant's head.

A certified orthotist utilized a patch to refer to landmarks including the glabella, opisthocranion, ears, occipital bone, orbital area, exocanthion, and tragion onto the infant's head, while the head was maintained in a neutral position (26). The anterior-posterior (A-P) and medio-lateral (M-L) diameters, and circumference at the apex of asymmetry were measured using computed metrics in Rodin4D software (Rodin4D, Eqwal group family, France). To compare the results before and after helmet therapy, the cranial vault asymmetry (CVA) and cranial vault asymmetrical index (CVAI) were computed and selected as reference measurements. The structural sensor scanner with a resolution of 1280960 pixels was used which takes 3 to 5 min to scan (Figure 2). Mesh mixer software (Autodesk company, United states) was used to clean up the scan.

### Helmet designing

After the scanning and measurements, the three-dimensional file was converted into Rodin4D-neo software format (Rodin4D, Eqwal group family, France) for rectification process. Meshmixer software (Autodesk company, United states) was used to create the trim lines that extend above the glabella to the inferior occipital bone above vertebra C2. ZBrush software was used to convert the mesh into a quad mesh so that it could be edited in CAD software.

### 3D helmet printing

Fusion 360 software (Autodesk company, United states) was utilized to generate the design of solid CAD for the helmet, with 4 mm (0.16 cm) thickness. Holes and padding measurements were added to the design. ZBrush software (Maxon company, German) was used to add personal features like pattern and text. Based on the necessary rigidity and flexibility of the helmet, the infill parameters were determined. The 3D design was sliced using the Cura Computer-Aided Manufacturing (CAM) software, which generated the instructions for the printer. The cube infill type was selected to provide the best impact resistance. The following printer settings were used; nozzle temperature 220° Celsius, bed temperature 60° Celsius, infill 85%, infill type cube, vase printing mood, direct extrusion, printing speed 30 mm (about 1.18 in)/S, and nozzle size 0.4 mm (about 0.02 inches). Printing was carried out using Anycubic Cobra Max printer (Anycubic, China, Shenzhen) and thermoplastic polyurethane (TPU) filament with hardness 98 Shore A. This material can apply gentle pressure after fronto-orbital advancement surgery, as there is no need for a stiffer material.

### Helmet fitting

**Figure 3:**
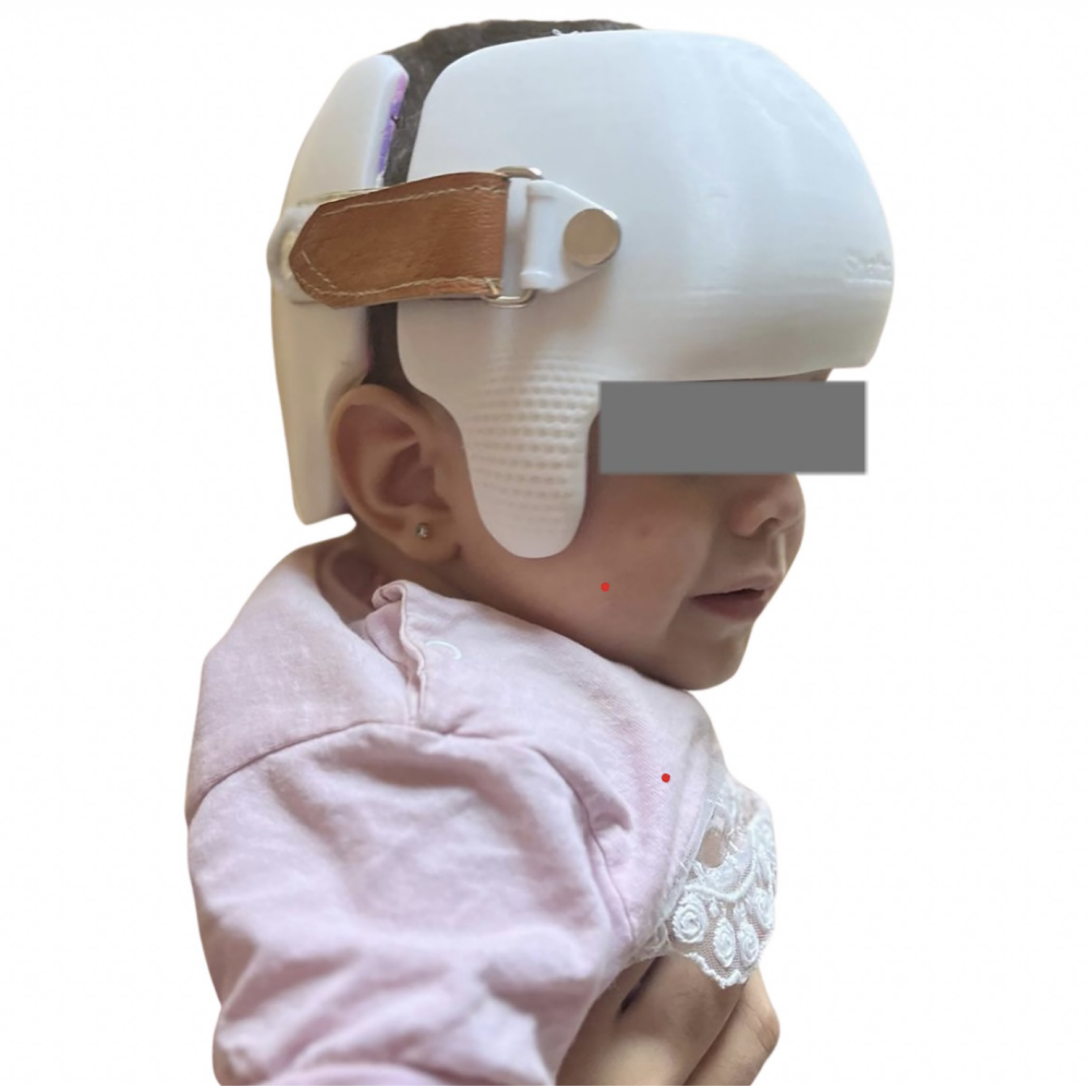
Infant fitted with a 3D-printed helmet.

After the initial check was successful, as shown in Figure 3. Checking any readiness was performed to assess the helmet's fitting. Pressure areas were assessed for pressure, friction or skin irritation. Afterwards, a strap was added, and 4 mm (about 0.16 in) of plastazote was applied for internal coverage to increase protection. Parents were advised to require their child to wear the helmet for approximately twenty-three hours per day. Bossing was controlled by pressure at the forehead. The helmet featured a posterior expansion area that would allow the bone to develop in that direction.

## Results

The patient had two follow-up appointments; the first one took place two months after the initial fitting. Parents did not report any difficulty in compliance, or side effects like bruises, lacerations or skin damage. There were no concerns of neurological or developmental impairments. Repeat cranial assessment was done and measurements were recorded (Table 1). The variations in diameters and circumferences demonstrate the growth pattern of the head. This is accompanied by a decrease in CVA and CVAI, which shows head shape correction and reduction of deformity. Craniosynostosis progressed from moderate to mild in severity. Three months later, at the second follow-up, The CVAI decreased from 7% to 2% and the CVA reduced from 7 mm to 3 mm. Given the improvement of head appearance supported by anthropometric measures, the outcomes of helmet therapy were considered more than satisfactory. Parents reported that they found the helmet comfortable and light weight and were satisfied with the results of the helmet therapy.

**Table 1:** Measurements before and after helmet therapy, at 2 and 5 months.

Measurements	Before helmet fitting	First follow up (2 months)	Second follow up (5 months)
Circumference at the apex	383 mm	419 mm	455 mm
Medio-lateral diameter	131 mm	142 mm	162 mm
Anterior-posterior diameter	106 mm	115 mm	132 mm
Diagonal line 1	113 mm	137 mm	127 mm
Diagonal line 2	107 mm	146 mm	125 mm
CVA	10 mm	6 mm	3 mm
CVAI	7%	5%	2%

## Discussion

The use of 3D printing technology is getting popular in medicine (27). Among broad range of 3D printed products, manufacturing of helmets using 3D printing technology is still uncommon in clinical practice. It has not become a standard of care; although there is a trend to adapt this technology among tech companies, manufacturers and commercial stakeholders (28–32). Food and Drug Authority (FDA) approvals have been obtained for 3D helmet manufacturing, which are largely based on non-clinical performance data (32, 33). It is interesting to observe that, in spite of commercial availability of these products, scientific data on clinical efficacy and outcomes is rarely documented (34). Similarly, data comparing safety and clinical efficacy of 3D printed helmet vs. molding helmets in treatment of paediatric skull deformities is also lacking.

The use of molding helmet is well documented for use of positional skull deformities such as plagiocephaly; while its use in metopic synostosis is mainly reported in relation to surgery (2). The findings of this study underscore the use of 3D helmet therapy and satisfactory reconstruction in a case of metopic craniosynostosis, after the endoscopy-assisted craniotomy surgical intervention. Our case had demonstrated the utilization of 3D-printed helmets following a surgical intervention appears to play a pivotal role in guiding and facilitating the natural skull reshaping of the infants. The personalized nature of these helmets, tailored to the unique cranial morphology of each patient, contributes significantly to the efficacy of the treatment by providing directed pressure to specific regions, thus promoting favourable growth patterns. The observed outcomes align with previous research highlighting the advantages of using 3D printing technology in the medical field (27, 34). By harnessing the precision and customization capabilities of this technology, clinicians can create comfortably fitting helmets, ensuring consistent pressure distribution without causing discomfort to the infant. Moreover, the ability to monitor and adjust the helmet design throughout the treatment period allows for dynamic adaptation to the changing needs of the patient, optimizing the therapeutic process.

The combination of precision and comfort in 3D-printed cranial helmets has been advocated as a transformative technology in the treatment of positional skull deformities. Conceptually, the principles of its use in single suture synostoses are not any different. Hence, it was considered to use this technology in an infant with metopic craniosynostosis. Molding helmets are typically made from rigid materials involving casting, rectification, and molding, which might not provide an optimal fit for every child. 3D printing allows for precision in creating helmets using scanning technologies to match the contours of an infant's head. This process is more comfortable to apply in infants as compared to the conventional process. The level of customization in 3D technology ensures a targeted pressure application, optimizing the corrective process, and preventing discomfort or skin related problems. Unlike molding helmets, 3D printed versions are often lighter and more breathable, which minimizes discomfort for infants. improving compliance with the recommended wear time. Consequently, this would enhance the effectiveness of the treatment. The prototyping and adjustments can be done quickly, offering early and timely interventions for patients. The positive outcomes of our case may be attributed to the benefits of 3D printed technology over conventional methods as mentioned above. Also, such a trial has not been attempted before and it opens an opportunity to consider 3D helmet technology when skull viability permits the treatment with conservative measures.

The standardization in manufacturing, printing, fabrication, application and maintenance of 3D printed medical devices could be a challenging process, especially in under resources places. Approvals from governing authorities may require a complex interplay of stakeholders who may not be well versed with this technology. Other than the hardware cost, financial aspects of licensure, software, staff training and maintenance contracts require formulation of policies and procedures to ensure a sustainable service. Various published guidelines may be used to advocate the need in a particular health setting. Since treatment of paediatric skull deformities require multidisciplinary services, institutional clinical management guidelines can help to ensure well formulated structure for orthotic use in cephalic disorders.

Published literature has largely emphasized the technical benefits, manufacturing accuracy and ease of application of 3D helmets (27, 28, 35–37); however, the outcome assessment should not only be limited to anthropometric measurements only. The skull deformities in infancy can be associated with neurodevelopmental delays and other neurological problems, which may manifest later in life (2, 38, 39). Since the helmet application is generally for months altogether, the changes in skull morphology occurs overtime. Similar to previous published literature on long term follow up using correction of cranial deformities with conventionally manufactured helmets (40), it is important to compare the long term outcomes with use of helmets manufactured using 3D printed technology. For neurocognitive or development delays which may demonstrate later in life, it may be difficult to determine if it was due to natural course of disease or helmet application or both. Also, outcomes of 3D helmet patients who already have neurological problems need to be studied as well. This renders the need of robust trials and scientific data to establish safety and clinical efficacy of use of 3D printed helmets.

## Conclusion

This study highlights the utilization of 3D printing technology in the treatment of metopic craniosynostosis after an endoscopy-assisted craniotomy surgical intervention. Helmet printed with 3D technology may offer a suitable treatment option for metopic craniosynostosis in selective cases. It also highlights the possibility of exploring 3D helmet therapy rather than molding helmets for metopic craniosynostosis. The clinical efficacy of 3D printed helmets in craniosynostosis need to be further explored. As advancements continue in both medical imaging and manufacturing technologies, the potential for further refinement and broader application of 3D-printed helmets in treating craniosynostosis is becoming increasingly viable. Long-term follow-up studies with larger sample sizes are imperative to comprehensively evaluate the durability, safety and clinical efficacy of 3D helmets in craniosynostosis.

## Limitations

The study identifies a novel application of 3D printing technology; however, the scope could benefit from a broader discussion of comparative outcomes with traditional molding helmets. Skull growth and reshaping in infants is a complex phenomenon, involving various innate pathophysiological processes. Attempts to reshape skull using 3D technology requires different use of materials and design as compared to molding helmets. Given the nature of this study as a single case report, the cause-effect relationship cannot be reliably concluded, and requires robust multicentre trials to test the observed relationship. Thus would restricts the generalizability of the findings. In this study, patient was followed up for short duration. Long term clinical outcomes other than anthropometric measures also need to be considered in reporting. Also, obtaining the parent feedback would be more scientific if it was taken through a questionnaire rather than verbally. Finally, potential challenges and limitations in implementing 3D printing technology in clinical settings include cost, accessibility, and regulatory hurdles.

## Data Availability

The original contributions presented in the study are included in the article/Supplementary Material, further inquiries can be directed to the corresponding author.
